# Nicotinamide-riboside shifts the differentiation of human primary white adipocytes to beige adipocytes impacting substrate preference and uncoupling respiration through SIRT1 activation and mitochondria-derived reactive species production

**DOI:** 10.3389/fcell.2022.979330

**Published:** 2022-08-22

**Authors:** Lilla Nagy, Boglárka Rauch, Tamás Szerafin, Karen Uray, Attila Tóth, Péter Bai

**Affiliations:** ^1^ Department of Medical Chemistry, Faculty of Medicine, University of Debrecen, Debrecen, Hungary; ^2^ Department of Cardiology and Heart Surgery, Faculty of Medicine, University of Debrecen, Debrecen, Hungary; ^3^ Section of Clinical Physiology, Department of Cardiology and Heart Surgery, Faculty of Medicine, University of Debrecen, Debrecen, Hungary; ^4^ HAS-UD Vascular Biology and Myocardial Pathophysiology Research Group, Hungarian Academy of Sciences, Budapest, Hungary; ^5^ Research Center for Molecular Medicine, Faculty of Medicine, University of Debrecen, Debrecen, Hungary; ^6^ MTA-DE Lendület Laboratory of Cellular Metabolism, Debrecen, Hungary; ^7^ MTA-DE Cell Biology and Signaling Research Group ELKH, Debrecen, Hungary

**Keywords:** beige adipocyte, mitochondrial oxidation, nicotinamide riboside, uncoupled respiration, adipocyte differentiation

## Abstract

Beige adipocytes play key roles in organismal energy and metabolic balance. In this study, we assessed whether the supplementation of human white adipocytes, differentiated from human adipose tissue-derived stem cells, with nicotinamide riboside (NR), a potent NAD + precursor, can shift differentiation to beige adipocytes (beiging). NR induced mitochondrial biogenesis and the expression of beige markers (TBX1 and UCP1) in white adipocytes demonstrating that NR can declutch beiging. NR did not induce PARP activity but supported SIRT1 induction, which plays a key role in beiging. NR induced etomoxir-resistant respiration, suggesting increases in the oxidation of carbohydrates, carbohydrate breakdown products, or amino acids. Furthermore, NR boosted oligomycin-resistant respiration corresponding to uncoupled respiration. Enhanced etomoxir and oligomycin-resistant respiration were dependent on mitochondrial reactive-species production. Taken together, NR supplementation can induce beiging and uncoupled respiration, which are beneficial for combatting metabolic diseases.

## 1 Introduction

Altered organismal energy homeostasis contributes to the induction of metabolic diseases, such as obesity (Hall et al., 2022). Adipocytes play a major role in organismal energy homeostasis by storing or oxidizing fatty acids (Giordano et al., 2014; Cohen and Spiegelman, 2016). Both brown and beige adipocytes have considerable oxidative phosphorylation (OXPHOS) capacity (Wu et al., 2012). White adipocytes are responsible for lipid storage and lipid clearance from the circulation. Uncoupling between the OXPHOS and ATP production yields heat in brown and beige cells due to the overexpression of uncoupling protein-1 (UCP1). Brown cells have multiple lipid droplets in the cytoplasm and are often called multilocular adipocytes. Brown cells enshroud major arteries in adults, and newborns have extra brown cell-rich adipose tissue localized in the interscapular region (Lidell et al., 2013; Nedergaard and Cannon, 2018). Unstimulated, resting beige adipocytes can be found in regular adipose tissues and have similar unilocular morphology as white adipocytes (Altshuler-Keylin et al., 2016). Without stimulation the expression of thermogenic genes is low in beige cells (Petrovic et al., 2010; Waldén et al., 2012; Shabalina et al., 2013). Beige cells induce mitochondrial biogenesis in response to adrenergic stimulus and are very efficient in fatty acid oxidation (Wu et al., 2012; Harms and Seale, 2013). Beige adipocyte dysfunction is a serious risk factor for developing obesity and type II diabetes (Claussnitzer et al., 2015; Alcala et al., 2019; Scherer, 2019).

NAD+ is a central molecule in biochemistry that is often referred to as the NAD + -node. NAD + has a redox cycle (NAD+ ↔ NADH) and a non-redox cycle in which NAD+ is cleaved into nicotinamide (NA) and ADP-ribose (ADPR) and then resynthesized (NAD+ ↔ NA + ADPR) (Houtkooper et al., 2010). NAD+ is cleaved by sirtuins (SIRTs), PARPs, and CD38, while the resynthesis involves members of the enzyme machinery of NAD + salvage (Houtkooper et al., 2010; Nikiforov et al., 2011; Cantó et al., 2013). Increases in NAD + levels induce pathways that upregulate mitochondrial biogenesis and, consequently, alleviate insulin resistance and obesity (Houtkooper et al., 2010; Nikiforov et al., 2011; Cantó et al., 2013). NAD + precursors can efficiently boost NAD + levels (Canto et al., 2012; Giroud-Gerbetant et al., 2019). NAD + metabolism is linked to adipocyte differentiation (Luo et al., 2017; Ryu et al., 2018; Huang et al., 2020; Szanto and Bai, 2020; Szanto et al., 2021)

Nicotinamide-riboside (NR) is an NAD + precursor that efficiently induces cellular NAD + levels and mitochondrial biogenesis (Canto et al., 2012). NR supplementation efficiently induced mitochondrial biogenesis in models of obesity (Canto et al., 2012; Jukarainen et al., 2016; Rappou et al., 2016; Jokinen et al., 2017; Asnani-Kishnani et al., 2019; Vannini et al., 2019; Nascimento et al., 2021), inflammatory diseases (Wu et al., 2022), Parkinson’s (Brakedal et al., 2022), non-alcoholic fatty liver disease (Dall et al., 2021), and aging (Sun et al., 2021). The objective of this study was to assess the effects of NR on the induction of shift of differentiation of white adipocytes to beige adipocytes (beiging) in a human adipose tissue-derived mesenchymal stem cell (hADMSC) model.

## 2 Materials and methods

### 2.1 Chemicals

Chemicals were purchased from Sigma-Aldrich (St. Louis, MO, United States) unless stated otherwise. NR was a generous gift from ChromaDex (Los Angeles, CA, United States). NR concentration was selected based on literature search [e.g., (Canto et al., 2012; Ryu et al., 2016)]. Mito-TEMPO, a mitochondrial antioxidant (Jankó et al., 2021; Kacsir et al., 2021) was from Sigma-Aldrich (St. Louis, MO, United States).

### 2.2 Ethical statement

The study protocol was approved by the Ethics Committee of the University of Debrecen (Hungary) and the National Medical Research Council Committee of Human Reproduction (ETT TUKEB). All experiments were carried out in accordance with the Declaration of Helsinki and the approved ethical guidelines and regulations. Written informed consent was obtained from all participants before the surgical procedure.

### 2.3 Isolation, culture, and differentiation of hADMSCs

Human ADMSCs, also called stromal-vascular fraction (SVF) cells, were isolated from pericardial adipose tissue specimens as described in (Kristof et al., 2015; Abdul-Rahman et al., 2016). The hADMSCs were maintained and differentiated to white or beige adipocytes as described in (Nagy et al., 2019). Primary human adipose tissue-derived stem cells (hADMSC) were cultured in DMEM F-12 HAM containing 10% FBS, 1% penicillin/streptomycin, 33 µM Biotion and 17 µM Pantothenic acid. Before the induction of differentiation cells were grown to confluency then the following media were applied. For the differentiation of white adipocytes during the 1st–3rd days of differentiation serum-free DMEM HAM-F12 supplemented with 1% Penicilline- streptomycin, 33 µM Biotin, 17 µM Pantothenic acid, 10 μg/ml Apotransferrin, 200 p.m. 3,3′,5-Triiodo-L-thyronine sodium salt, 20 nM Human Insulin, 100 nM Hydrocortisone, 2 µM Rosiglitazone, 25 nM Dexamethasone, 500 µM 3-Isobutyl-1-methylxanthine that is exchanged for serum-free DMEM HAM-F12 supplemented with 1% Penicilline- streptomycin, 33 µM Biotin, 17 µM Pantothenic acid, 10 μg/ml Apotransferrin, 200 p.m. 3,3′,5-Triiodo-L-thyronine sodium salt, 20 nM Human Insulin, 100 nM Hydrocortisone between the 4th–14th day of differentiation. For the induction of the differentiation of beige adipocytes serum-free DMEM HAM-F12 supplemented with 1% Penicilline- streptomycin, 33 µM Biotin, 17 µM Pantothenic acid, 10 μg/ml Apotransferrin, 200 p.m. 3,3′,5-Triiodo-L-thyronine sodium salt, 850 nM Human Insulin, 1 µM Dexamethasone, 500 µM 3-Isobutyl-1-methylxanthine was used. From day 4^th^ to day 14 serum-free DMEM HAM-F12 supplemented with 1% Penicilline- streptomycin, 33 µM Biotin, 17 µM Pantothenic acid, 10 μg/ml Apotransferrin, 200 p.m. 3,3’,5-Triiodo-L-thyronine sodium salt, 850 nM Human Insulin and 500 nM Rosiglitazone was applied. Medium was changed every 2 days. A subset of white adipocytes was treated with 500 µM NR for 14 days during the differentiation process.

### 2.4 Immunofluorescence and confocal microscopy

Mitochondrial structure was determined by staining differentiated hADMSCs with TOMM20 immunohistochemistry similar to (Jankó et al., 2021). The hADMSCs were seeded on glass coverslips and differentiated as described in 2.3. To detect TOMM20, differentiated cells were washed with PBS, fixed with 4% paraformaldehyde for 10 min at 37°C, and permeabilized with 1% Triton X-100 in PBS for 10 min. Between each step, cells were rinsed twice with PBS. Cells were blocked with 1% bovine serum albumin (BSA) in PBS for 1 h at room temperature. TOMM20 primary antibody was applied overnight (4°C, humidified chamber, diluted in blocking buffer). The next day, cells were washed and probed with Alexa Fluor 647-conjugated secondary antibody (Goat anti-Mouse IgG (H + L), Thermo Fisher Scientific, Waltham, MA, United States; excitation: 651 nm, emission: 667 nm). The pictures were taken with the ×40 objective of the system. Cell nuclei were visualized with DAPI (NucBlue Fixed Cell ReadyProbes Reagent, Thermo Fisher Scientific, Waltham, MA, United States). Confocal images were acquired with a Leica TCS SP8 confocal microscope (Leica, Wetzlar, Germany) and LAS X 3.5.5.19976 software (Leica, Wetzlar, Germany). Nonspecific binding of secondary antibodies was checked in control experiments (not shown). Processed images were analyzed using the ImageJ software Mito-Morphology Macro (Dagda et al., 2009; Dagda, 2019). Mitochondrial content, perimeter, circularity, and form factor were calculated from confocal microscopic images.

### 2.5 Gene expression and RT-qPCR

Reverse transcription-coupled real-time quantitative PCR (RT-qPCR) reactions were performed as described in (Bai et al., 2007). Primers are summarized in Table 1. Expression was normalized to the geometric mean of β-actin and 36B4 genes and was expressed as fold change.

**TABLE 1:** Human primers used in RT-qPCR reactions.

Gene	Forward	Reverse
36B4	5′-CCA​TTG​AAA​TCC​TGA​GTG​ATG​TG-3′	5′-GTC​GAA​CAC​CTG​CTG​GAT​GAC-3′
β-actin	5′-GAC​CCA​GAT​CAT​GTT​TGA​GAC​C-3′	5′-CAT​CAC​GAT​GCC​AGT​GGT​AC -3′
UCP1	5′-AAC​GAA​GGA​CCA​ACG​GCT​TTC-3′	5′-GGC​ACA​GTC​CAT​AGT​CTG​CCT​TG -3′
TBX1	5′-TCC​CAC​CTT​CCA​AGT​GAA​GCT​C -3′	5′-CAC​GAT​TTG​CTT​CAT​CCA​CTG​C -3′
PRDM16	5′-CAC​TGT​GCA​GGC​AGG​CTA​AGA​A-3′	5′-AGA​GGT​GGT​TGA​TGG​GGT​GAA​A-3′
COX7A1	5′-ATA​CGG​AAA​CAG​GCT​CGG​AGG​T-3′	5′-ATC​CGT​TTC​GGT​CTC​GGA​ATT​T-3′
CIDEA	5′-TCT​CCA​ACC​ATG​ACA​GGA​GCA​G-3′	5′-AAT​GCG​TGT​TGT​CTC​CCA​AGG​T-3′
TMEM26	5′-ACC​TCC​CAT​GTG​TGG​ACA​TCC​T-3′	5′-ACC​AAC​AGC​ACC​AAC​AAC​CTC​A -3′
SIRT1	5′-TGG​CAA​AGG​AGC​AGA​TTA​GTA​GGC-3′	5′-TGG​ACT​CTG​GCA​TGT​CCC​ACT-3′
PGC1α	5′-TTC​CTC​TGA​CCC​CAG​AGT​CAC​C-3′	5′-TTG​CAA​GAG​GAC​TTC​AGC​TTT​GG-3′
PPARγ1	5′-GTG​GCC​GCA​GAT​TTG​AAA​GAA​G-3′	5′-CCA​TGG​TCA​TTT​CGT​TAA​AGG​CTG-3′
PPARγ2	5′-CAG​CAA​ACC​CCT​ATT​CCA​TGC-3′	5′-GGG​AGT​GGT​CTT​CCA​TTA​CGG-3′
ADIPOQ	5′-TTA​AAA​CCT​CCC​CCA​AGC​AGA-3′	5′-GCC​TTG​AGG​AAC​AGG​GAT​GAG-3′
FAS	5′-GCA​GGA​GCT​CAA​GAA​GGT​GAT​C-3′	5′-ACC​AGG​TTG​TTG​ACA​TTG​TAC​TCG-3′
FABP4	5′-GGA​AAG​TCA​AGA​GCA​CCA​TAA​CC-3′	5′-GCT​CTC​TCA​TAA​ACT​CTC​GTG​GAA​G-3′
HSL	5′-GAA​GCC​TTT​GAG​ATG​CCA​CTG-3′	5′-CTC​ACT​GTC​CTG​TCC​TTC​ACG-3′
leptin	5′-CAC​ACA​CGC​AGT​CAG​TCT​CCT​C-3′	5′-GTA​TGC​CTT​CCA​GAA​ACG​TGA​TCC-3′
LPL	5′-CTG​GAT​GGA​GGA​GGA​GTT​TAA​CTA​CC-3′	5′-CTG​CAT​CAT​CAG​GAG​AAA​GAC​G-3′
PLIN1/2	5′-GAA​CAA​GTT​CAG​TGA​GGT​AGC​AGC-3′	5′-CTT​GGT​TGA​GGA​GAC​AGC​AGG-3′
TNFa	5′-GCA​GTC​AGA​TCA​TCT​TCT​CGA​AC-3′	5′-GAA​GAG​GAC​CTG​GGA​GTA​GAT​GAG-3′
PARP1	5′-CAC​TGG​TAC​CAC​TTC​TCC​TGC​TTC-3′	5′-CTT​TGC​CTG​TCA​CTC​CTC​CAG-3′
PARP2	5′-GCT​AAA​TCA​GAC​CAA​TCT​CC-3′	5′-CAG​GCT​GTG​CTG​TCC​CAT​TT-3′
PARP3	5′- CTT​CCT​GGG​CCT​CAT​CCT​CTG-3′	5′- CAA​CCG​CTT​CTT​CAC​CTG​CTG-3′
PARP5a	5′- AAC​ATC​CTT​CCT​TCC​AAA​ACC​T-3′	5′- GGC​AAA​CGT​AAA​TGC​AAA​GG-3′
PARP5b	5′- AAG​GTT​ACC​CGG​CAA​AAG​A-3′	5′- TGG​GTG​TCC​AGT​TCA​CAA​AG-3′
PARP10	5′-CTG​TGG​ACC​TGC​TGT​TGC​TG-3′	5′-GGA​TGT​CGT​AGT​GGG​GGA​CA-3′

### 2.6 Protein extraction and western blotting

The hADMSCs were seeded, differentiated, and treated in 10 cm Petri dishes. Cell were rinsed with PBS 2 times, scraped, centrifuged, and lysed in RIPA lysis buffer (50 mM Tris, 150 mM NaCl, 0.1% SDS, 1% Triton X 100, 0.5% sodium deoxycholate, 1 mM EDTA, 1 mM Na3VO4, 1 mM NaF, and protease inhibitor cocktail). Western blotting was performed as described by (Nagy et al., 2018). Blots were probed with the antibodies summarized in Table 2. Signals were detected using enhanced chemiluminescence (ECL) and were captured by ChemiDoc Touch (Bio-Rad Laboratories, CA, United States).

**TABLE 2:** Primary antibodies used in the study.

Target	Type	Company	Dilution
TOMM20	monoclonal	Abcam, Cambridge UK	1:200
UCP1	monoclonal	Cell Signaling, Danvers MA, United States	1:1000
TBX1	polyclonal	GeneTex, Irvine, CA, United States	1:500
PGC1a	polyclonal	Thermo Fisher Scientific, Waltham, MA, United States	1:1000 for WB
			1:200 for IP
acetyl-lysine antibody	polyclonal	Cell Signaling, Danvers MA, United States	1:500
Poly (ADP-ribose) (10H)	monoclonal	Sigma aldrich	1:500
Mono(ADP-ribose)	monoclonal	Sigma aldrich	1:1000
β-Actin−Peroxidase	polyclonal	Sigma aldrich	1:20000

### 2.7 Determination of differentiation rate

The rate of differentiation was determined as described in (Bai et al., 2007). The hADMSC cells were seeded, differentiated, and treated in 24-well plates. On the day of the experiment, cells were stained with Nile Red dye (10 μg/ml) in medium and incubated for 30 min at 37°C. Cells were rinsed with PBS 3 times, digested with Trypsin-EDTA, suspended in PBS, and pipetted in FACS tubes. The differentiation rate was determined using a NovoCyte Flow Cytometer (NovoCyte 3000, Acea Biosciences Inc., San Diego, United States) and analyzed using NovoExpress 1.2.5 Software. Differentiation rate was expressed as a percent of all cells.

### 2.8 Determination of oxygen consumption

Oxygen consumption rate (OCR) was determined using an XF96 Flux Analyzer using the assay plates designed for the instrument (Agilent Technologies, CA, United States). The hADMSCs were seeded in 96-well assay plates, then differentiated as described above. After recording the baseline oxygen consumption, cells were treated with a single bolus dose of dibutyril-cAMP (500 μM final concentration) to simulate adrenergic stimulation and OCR was recorded in 30 min intervals 5 times. Next, etomoxir (50 μM final concentration) was applied and OCR was recorded every 5 times for 3 min. Etomoxir is an inhibitor of mitochondrial fatty acid import (Declercq et al., 1987), etomoxir-sensitive respiration corresponds to fatty acid oxidation, while etomoxir-resistant respiration corresponds to the oxidation of other, non-fatty acid substrates. The cells were then treated with oligomycin (2.5 μM final concentration), and OCR was recorded every 5 times for 3 min. Oligomycin blocks the F1/F0 ATP synthase. Therefore, etomoxir-resistant respiration corresponds to uncoupled respiration. Finally, cells were treated with a single bolus dose of antimycin A (10 μM) and rotenone (5 μM) and OCR was recorded every 5 times for 3 min. These drugs completely block mitochondrial respiration and can be used to determine the baseline fluorescence intensity (i.e., background). After the measurement, XF96 cell plates were stained with Nile Red dye, and cell number and differentiation rate were determined using a Novocyte Flow cytometer (NovoCyte 3000, Acea Biosciences Inc., San Diego, United States) and analyzed using NovoExpress 1.2.5 Software. OCR values were normalized to the differentiation rate for each well and normalized readings were analyzed and plotted. The calculation and measurement procedure are published in (Miko et al., 2017).

### 2.9 Immunoprecipitation

Cells were lysed in RIPA lysis buffer as described in 2.6. PGC1α acetylation levels were analyzed by immunoprecipitating lysates with anti-PGC1α antibodies followed by Western blotting using an acetyl-lysine antibody and normalization to total PGC1α levels similar to (Bai et al., 2011a; Bai et al., 2011b).

### 2.10 Statistical analysis

Data were analyzed using GraphPad Prism nine software. The modified Thompson Tau test was used to identify outlier data points that were removed from the analysis. Normality was tested using D’Agonstino and Pearson tests. Statistical tests are stated in the figure legends. All data is represented as average ±SD, unless stated otherwise. All experiments were repeated at least three times.

## 3 Results

### 3.1 NR treatment induces mitochondrial biogenesis and mitochondrial oxidation in human primary white adipocytes

**FIGURE 1:**
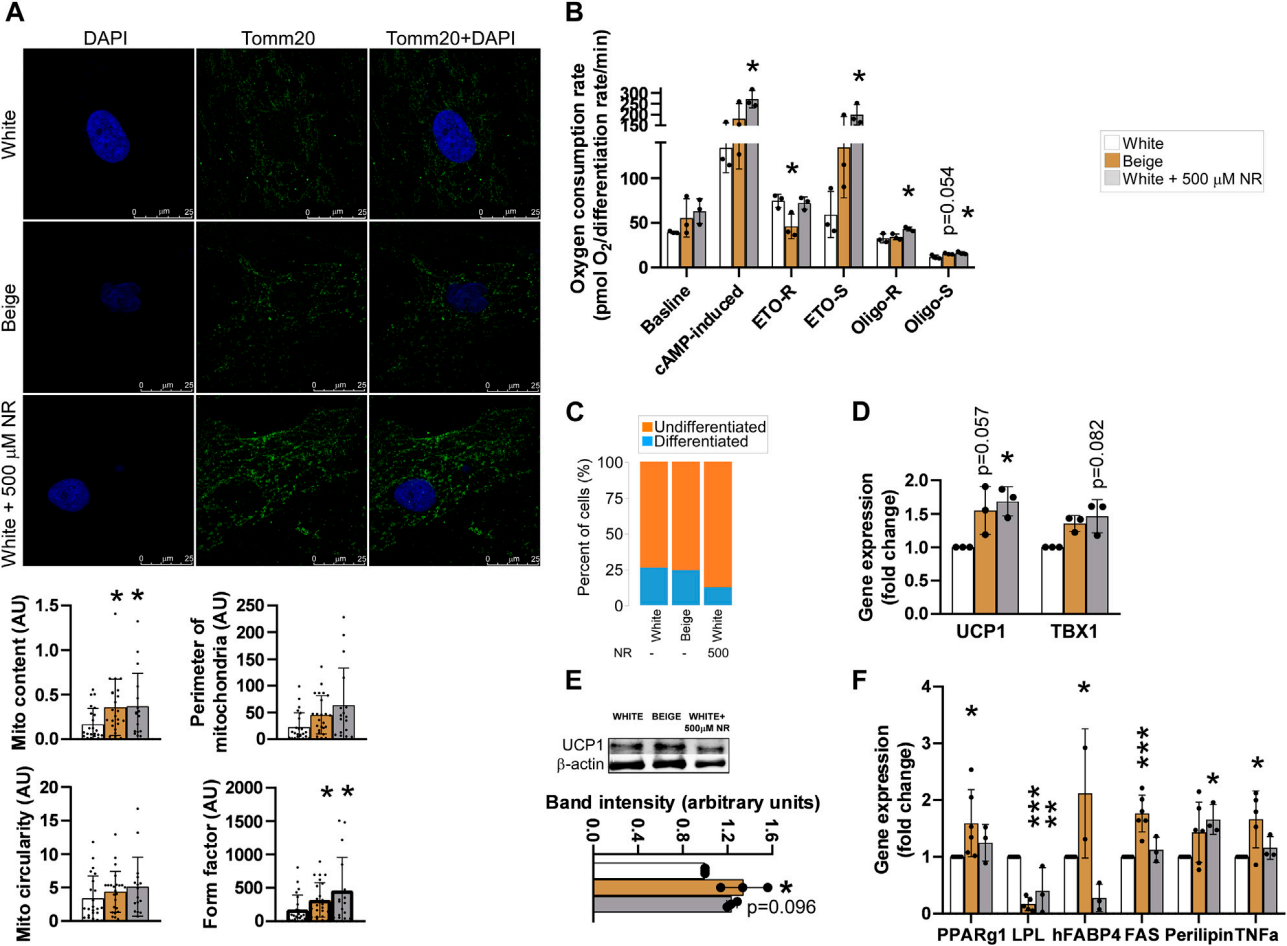
NR-treatment shifts the differentiation of white adipocytes to beige-like cells. The hADMSCs cells from three donors were seeded and differentiated to mature adipocytes. Cells were treated with NR (500 µM) throughout the differentiation process. **(A)** Differentiated cells were stained with TOMM20 antibody, then mitochondrial quantity and morphology were evaluated **(B)** Human adipose tissue-derived mesenchymal stem cells were seeded into Seahorse assay plates and assayed after differentiation. Mitochondrial oxygen consumption was assessed as described in Materials and Methods. **(C)** Adipocyte differentiation rate was determined as described in Materials and Methods **(D)** The expression levels of the indicated genes were measured by RT-qPCR in differentiated human adipose tissue-derived mesenchymal stem cells. **(E)** UCP1 protein expression was measured by Western blot in differentiated human adipose tissue-derived mesenchymal stem cells **(F)** The expression levels of the indicated genes were measured by RT-qPCR in differentiated human adipose tissue-derived mesenchymal stem cells. Normality was checked. Statistical significance was assessed by One-way ANOVA test followed by a post-hoc test versus white adipocytes. *, **, *** indicate significant differences between groups at *p* < 0.05, *p* < 0.01 or *p* < 0.001, respectively. Data are represented as means ± SD. Data are expressed as fold change normalized to white adipocytes. Abbreviations: ETO-S, etomoxir sensitive; ETO-R, etomoxir-resistant; hADMSC, human adipose tissue-derived mesenchymal stem cell; NR, nicotinamide-riboside; PAR, poly (ADP-ribose); UCP, uncoupling protein-1.

First, we measured mitochondrial content by immunofluorescently labeling TOMM20, a mitochondrial marker protein, followed by image analysis. Mitochondrial content was higher in beige cells compared with white adipocytes, similar to NR-treated white adipocytes (Figure 1A). Furthermore, beige and NR-treated adipocytes had a more fused mitochondrial network compared with the network of white adipocytes, marked by increases in the form factor (Figure 1A). These changes resulted in increases in mitochondrial oxidative activity upon cAMP stimulation. (Figure 1B). Furthermore, the substrates for mitochondrial oxidation were altered. In beige adipocytes, etomoxir-resistant respiration, representing carbohydrate and amino acid oxidation, decreased, while etomoxir-sensitive respiration, representing fatty acid oxidation, increased (Figure 1B) compared with respiration in white adipocytes. In NR-treated cells, etomoxir-resistant respiration was similar to white adipocytes, while, fatty acid oxidation was higher in beige adipocytes (Figure 1B), highlighting the robust increases in fatty acid oxidation in response to NR treatment. Oligomycin-resistant respiration, a proxy for uncoupled respiration, increased in beige adipocytes compared with white and beige cells (Figure 1B). Furthermore, oligomycin-sensitive respiration, representing coupled-respiration, increased both in beige and NR-treated cells (Figure 1B).

NR treatment reduced the rate of differentiation in adipocytes compared with white adipocytes (Figure 1C), similar to treatment of cells with olaparib, a PARP inhibitor (Nagy et al., 2019). The mRNA and protein expression levels of a brown and beige marker gene, *uncoupling protein-1* (*UCP1*), which drives uncoupled respiration and heat generation (Cinti, 2017), and *T-Box Transcription Factor* (*TBX1*), a beige-specific marker. The mRNA expression levels of *UCP1* and *TBX1* were higher in beige and NR-treated cells compared with white adipocytes (Figure 1D). Furthermore, higher UCP1 mRNA expression was translated to higher UCP1 protein levels in beige and NR-treated cells (Figure 1E). Finally, we assessed the mRNA expression of adipogenic marker genes. The expression of peroxisome proliferator activated receptor- γ1 (PPARγ1)*,* fatty acid binding protein-4 (FABP4), fatty acid synthase (FAS), perilipin, and tumor necrosis factor α (TNFα) increased, while lipoprotein lipase (LPL) decreased in beige adipocytes compared with expression levels in white adipocytes (Figure 1F). NR-treatment of white adipocytes did not elicit identical changes to gene expression as beige differentiation. NR treatment induced the expression of *perilipin* and decreased the expression of *LPL* and *FABP4* but did not alter the expression of *PPARγ*, *FAS,* and *TNFα,* suggesting that NR-treated cells have a different adipogenic enzyme composition and, therefore, different function.

### 3.2 NR treatment induces SIRT1 activation but not excess PARP1 activation

**FIGURE 2:**
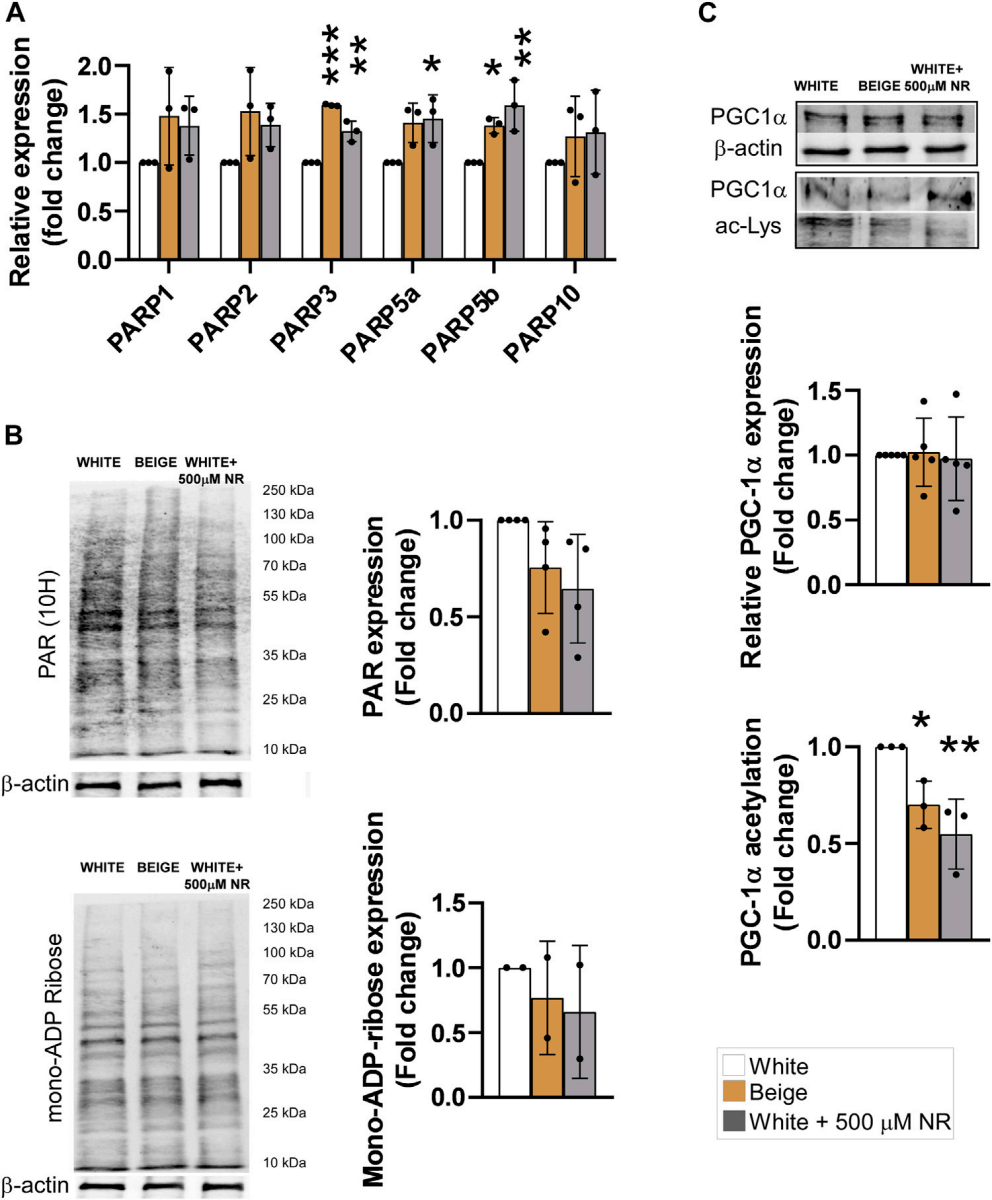
NR supplementation does not induce PARP activity but induces SIRT1 in hADMSC-derived adipocytes. Human adipose tissue-derived mesenchymal stem cells from three different controls were differentiated to adipocytes as described in Materials and Methods. **(A)** The expression levels of the indicated genes were determined using RT-qPCR **(B)** Poly (ADP-ribose) and mono-ADP-ribose levels were determined by Western blot. **(C)** PGC-1α was immunoprecipitated and acetylation levels were determined in the immunprecipitates. Normality was checked. Statistical significance was assessed by One-way ANOVA test followed by a post-hoc test versus white adipocytes. *, **, *** indicate significant differences between groups at *p* < 0.05, *p* < 0.01 or *p* < 0.001, respectively. Data are represented as means ± SD. Data are expressed as fold change normalized to white adipocytes. Abbreviations: hADMSC, human adipose tissue-derived mesenchymal stem cell; NR, nicotinamide-riboside; PAR, poly (ADP-ribose); PGC1α, peroxisome proliferator-activated receptor gamma coactivator-1α.

NR is a precursor of NAD + salvage and can support the activity of NAD + -dependent enzymes, such as PARPs or sirtuins (Canto et al., 2012). First, we assessed the mRNA expression of PARP enzymes known to be involved in regulating mitochondrial metabolism, including PARP1 (Virag et al., 1998; Bai et al., 2011b), PARP2 (Bai et al., 2011a; Mohamed et al., 2014), PARP3 (Rodriguez-Vargas et al., 2020), PARP5a (TNKS1), PARP5b (TNKS2) (Yeh et al., 2009; Wang et al., 2020), and PARP10 (Marton et al., 2018). The activity of PARP1 and PARP2, the enzymes responsible for the bulk of cellular PARP activity (Schreiber et al., 2002; Szanto et al., 2011), and PARP10 did not change in response to NR supplementation (Figure 2A). However, the mRNA levels of PARP3, PARP5a, and PARP5b were slightly increased by NR (Figure 2A). Of note, the mRNA expression levels of PARP3, PARP5a, and PARP5b were also elevated upon differentiation of hADMSCs into beige adipocytes (Figure 2A). These findings prompted us to assess poly- and mono-ADP-ribosylation in cells. No differences in cellular levels of poly-ADP-ribose or mono-ADP-ribose were detected (Figure 2B), suggesting that NR supplementation did not induce PARP activity.

Sirtuins are also major NAD + consumers in cells and SIRT1 is a major driver of beige differentiation (Fu et al., 2014; Khanh et al., 2018; Liao et al., 2021). Therefore, we assessed SIRT1 activity in differentiated cells. Peroxisome proliferator-activated receptor gamma coactivator-1α (PGC1α) is a target of SIRT1 deacetylation (Nemoto et al., 2005). Hence, determining changes in PGC1α acetylation is a good proxy for SIRT1 activity. In addition, PGC1α is a key element in beige differentiation (Yan et al., 2016). PGC1α acetylation levels decreased in beige cells compared with white adipocytes, similar to NR-treated cells (Figure 2C), suggesting the SIRT1 activity increased during beige differentiation and upon NR-induced shift in differentiation.

### 3.3 NR treatment supports uncoupled respiration through mitochondrial reactive species production

**FIGURE 3:**
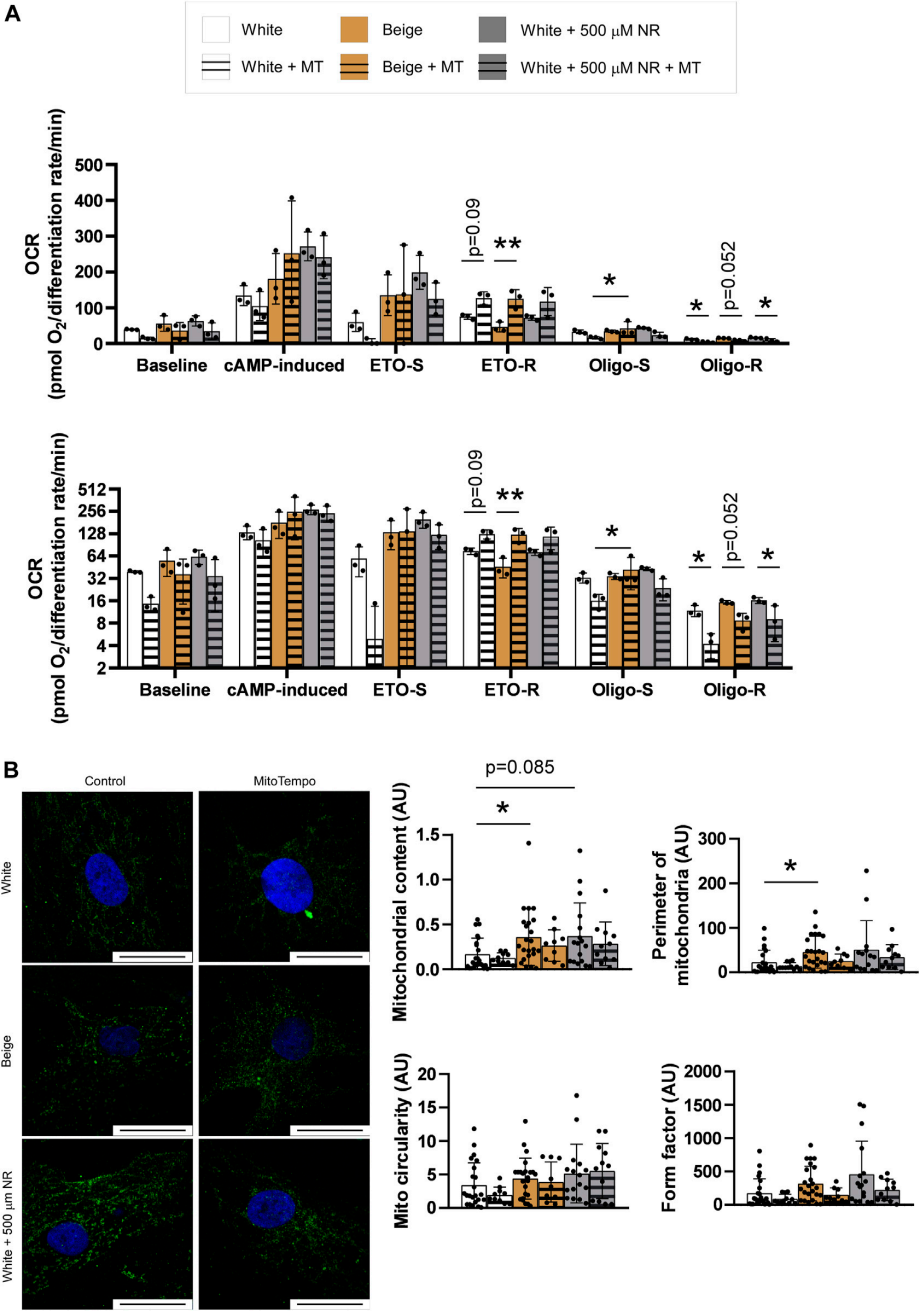
Mitochondria-derived reactive species production supports a switch towards uncoupled respiration. **(A)** Human adipose tissue-derived mesenchymal stem cells from three different donors were seeded in Seahorse plates and differentiated to adipocytes and mitochondrial oxidation was determined as described in Materials and Methods. The bottom graph depicts the same data on a log2-scale for better visibility **(B)** Human adipose tissue-derived mesenchymal stem cells from three different donors were seeded on coverslips, differentiated, stained with a TOMM20 antibody, and mitochondrial morphology was assessed as described in Materials and Methods. The bar equals to 25 µm. Normality was checked. Statistical significance was assessed by Two-way ANOVA test followed by a post-hoc test that compares all possible combinations. * and ** symbolize significant differences between groups at *p* < 0.05 or *p* < 0.01, respectively. Data are represented as means ± SD. Data are expressed as fold change, where white adipocytes were considered as 1. Abbreviations: ETO-S, etomoxir sensitive; ETO-R, etomoxir-resistant; hADMSC, human adipose tissue-derived mesenchymal stem cell; Mito, mitochondria; MT, Mito-TEMPO; NR, nicotinamide-riboside; Oligo-S, oligomycin sensitive; Oligo-R, oligomycin resistant.

Mitochondrial reactive species play a fundamental role in inducing mitochondrial biogenesis (Valero, 2014; Fu et al., 2018; Ryoo and Kwak, 2018; Palmeira et al., 2019; Jankó et al., 2021). We tested whether reactive species were produced in our system by supplementing the differentiation medium with Mito-TEMPO, a mitochondrial-targeted antioxidant, during the differentiation process. Mito-TEMPO treatment did not influence baseline, cAMP-induced, or etomoxir-sensitive (representing fatty acid oxidation) mitochondrial oxidation rates (Figure 3A). However, etomoxir-resistant rates (representing amino acid and carbohydrate oxidation) were increased in response to Mito-TEMPO treatment in white and beige adipocytes, suggesting that reactive species production is important in driving fatty acid oxidation (Figure 3A). A similar trend was observed in NR-treated cells; however, the changes were not statistically significant (Figure 3A). No differences in the oligomycin-sensitive fraction of mitochondrial oxidation, corresponding to coupled-respiration, were detected. Nevertheless, Mito-TEMPO suppressed oligomycin-resistant (uncoupled) respiration in white and beige adipocytes and NR-treated white adipocytes (Figure 3A). These results highlight the key role of reactive species production in uncoupling. Mito-TEMPO treatment did not influence mitochondrial morphology (Figure 3B).

## 4 Discussion

In this study, we assessed the applicability of an NAD + precursor, NR, in shifting the differentiation of human adipose-derived pluripotent cells differentiated to white adipocytes. NR supplementation induced mitochondrial biogenesis and, consequently, increased mitochondrial oxidative capacity. These changes coincided with increased expression of UCP1, a marker of uncoupled mitochondrial oxidation, and TBX1, a beige marker gene. NR suppressed the rate of differentiation, similar to olaparib, a PARP inhibitor that induces NAD + -sparing and declutches beige transdifferentiation in the same model system (Nagy et al., 2019).

Previous studies identified NAD + as a key player in the induction of thermogenesis (Yamaguchi et al., 2019) and NR supplementation induces NAD + levels (Canto et al., 2012). NAD + interacts with a plethora of enzymes (Ziegler, 2000; Houtkooper et al., 2010; Nikiforov et al., 2011; Chiarugi et al., 2012; Bai et al., 2015), some of which are involved in intermediary metabolism and higher order metabolic regulation in beige or brown adipose tissue differentiation and function. These enzymes include AMP-activated protein kinase (AMPK) (Shan et al., 2013; Abdul-Rahman et al., 2016; Mottillo et al., 2016; Zhu et al., 2016; Desjardins and Steinberg, 2018; Imran et al., 2018; Wu et al., 2018; Serrano et al., 2020), PGC1α (Shan et al., 2013; Nagy et al., 2019), and SIRT1 (Qiang et al., 2012; Khanh et al., 2018; Asnani-Kishnani et al., 2019). Serrano and colleagues showed that NR also induces epigenetic changes (Serrano et al., 2020). We observed the activation of SIRT1 in response to NR treatment, which is consistent with previous studies (Qiang et al., 2012; Khanh et al., 2018). PARP enzymes are major consumers of NAD+ and can degrade NAD+ and limit NAD + availability to sirtuins (Bai et al., 2011a; Bai et al., 2011b; Cantó et al., 2013). However, we did not detect PARP activation in response to NR supplementation, suggesting that increases in NAD + support SIRT1 but not activation of PARP enzymes.

We observed changes in substrate preferences in NR-treated cells that were dependent on mitochondria-derived reactive species. Mito-TEMPO, a mitochondrial reactive species scavenger, induced etomoxir-resistant respiration in adipocytes but did not affect etomoxir-sensitive respiration (Figure 3A). Etomoxir is an inhibitor of mitochondrial fatty acid import (Declercq et al., 1987). Hence, etomoxir-sensitive respiration corresponds to fatty acid oxidation, while etomoxir-resistant respiration corresponds to the oxidation of carbohydrates, carbohydrate degradation products (e.g., pyruvate), and other substrates (e.g., amino acids). Thus, our data suggest that increased mitochondrial oxidation upon cAMP stimulation is dependent on increased carbohydrate and amino acid oxidation. Our observations are consistent with Dall et al. (Dall et al., 2019), who showed that the beneficial effects of NR treatment in liver mitochondria were dependent on glutamine and pyruvate oxidation. Pyruvate oxidation was implicated in modulating obesity and adipose tissue function (Ingram and Roth, 2021). However, our findings conflict with the findings of Canto et al. (Canto et al., 2012), who reported increases in fatty acid oxidation in NR-treated C57/Bl6 mice. Shi et al. (Shi et al., 2017) reported that NR administration to mice supported metabolic flexibility marked by large changes in respiratory quotient (or respiratory exchange ratio) values between the fed and fasted states compared with vehicle-fed animals.

Another important finding of this study is the key role that mitochondria-derived reactive species play in setting the ratio of coupled to uncoupled respiration. Mito-TEMPO treatment decreased the oligomycin-resistant fraction of respiration, indicating decreased uncoupled respiration in white, beige, and NR-treated adipocytes, while Mito-TEMPO had no effect on oligomycin-sensitive, coupled respiration. In other words, mitochondrial reactive species production is needed to support uncoupled respiration. Consistent with this conclusion, Chouchani and colleagues showed that the addition of the general thiol reductant, N-acetyl-cysteine (NAC), reduced UCP1-mediated uncoupling in mitochondria (Chouchani et al., 2016). NAC is a general thiol reducing agent and may affect thiols and redox-labile groups outside the mitochondria. Mito-TEMPO is specific for the mitochondria. Hence, our results consolidate the role of mitochondrial reactive species production in inducing uncoupled respiration. In addition, these data highlight the role of reactive species in regulating mitochondrial oxidation. The accepted view is that reactive species inhibit mitochondrial respiration by oxidizing components of the electron transport chain (Wang et al., 2010). Our results indicate that reactive species overproduction can induce uncoupling that spares destructive oxidation of mitochondrial oxidative phosphorylation machinery. Nevertheless, in human subjects, NR had no impact on thermogenesis (Nascimento et al., 2021), leaving the question open about how these findings can be translated to humans.

In this study, we showed that the application of NR to hADMSCs shifted the differentiation of white adipocytes to beige. Furthermore, we showed that SIRT1 induction and reactive species production play key roles in differentiation, mitochondrial biogenesis, substrate preference, and the induction of uncoupled respiration. These results have implications for understanding organismal energy balance mechanisms and may have implications in the metabolic arena.

## Data Availability

The datasets presented in this study can be found in online repositories. The names of the repository/repositories and accession number(s) can be found below: https://figshare.com/s/b553ecd08aca5c0b1347.
